# Functional network connectivity patterns predicting the efficacy of repetitive transcranial magnetic stimulation in the spectrum of Alzheimer’s disease

**DOI:** 10.1186/s41747-023-00376-3

**Published:** 2023-10-24

**Authors:** Haifeng Chen, Mengyun Li, Zhiming Qin, Zhiyuan Yang, Tingyu Lv, Weina Yao, Zheqi Hu, Ruomeng Qin, Hui Zhao, Feng Bai

**Affiliations:** 1 Department of Neurology, Nanjing Drum Tower Hospital, Affiliated Hospital of Medical School, Nanjing University, 321 Zhongshan Road, Nanjing, 210008 Jiangsu China; 2 Nanjing Drum Tower Hospital Clinical College of Traditional Chinese and Western Medicine, Nanjing University of Chinese Medicine, Nanjing, China; 3 Jiangsu Key Laboratory of Molecular Medicine, Medical School of Nanjing University, Nanjing, China; 4 Jiangsu Province Stroke Center for Diagnosis and Therapy, Nanjing, China; 5 Nanjing Neuropsychiatry Clinic Medical Center, Nanjing, China; 6 Geriatric Medicine Center, Affiliated Hospital of Medical School, Taikang Xianlin Drum Tower Hospital, Nanjing University, Nanjing, China

**Keywords:** Alzheimer’s disease, Brain, Machine learning, Magnetic resonance imaging, Transcranial magnetic stimulation

## Abstract

**Background:**

Neuro-navigated repetitive transcranial magnetic stimulation (rTMS) is potentially effective in enhancing cognitive performance in the spectrum of Alzheimer’s disease (AD). We explored the effect of rTMS-induced network reorganization and its predictive value for individual treatment response.

**Methods:**

Sixty-two amnestic mild cognitive impairment (aMCI) and AD patients were recruited. These subjects were assigned to multimodal magnetic resonance imaging scanning before and after a 4-week stimulation. Then, we investigated the neural mechanism underlying rTMS treatment based on static functional network connectivity (sFNC) and dynamic functional network connectivity (dFNC) analyses. Finally, the support vector regression was used to predict the individual rTMS treatment response through these functional features at baseline.

**Results:**

We found that rTMS at the left angular gyrus significantly induced cognitive improvement in multiple cognitive domains. Participants after rTMS treatment exhibited significantly the increased sFNC between the right frontoparietal network (rFPN) and left frontoparietal network (lFPN) and decreased sFNC between posterior visual network and medial visual network. We revealed remarkable dFNC characteristics of brain connectivity, which was increased mainly in higher-order cognitive networks and decreased in primary networks or between primary networks and higher-order cognitive networks. dFNC characteristics in state 1 and state 4 could further predict individual higher memory improvement after rTMS treatment (state 1, *R* = 0.58; state 4, *R* = 0.54).

**Conclusion:**

Our findings highlight that neuro-navigated rTMS could suppress primary network connections to compensate for higher-order cognitive networks. Crucially, dynamic regulation of brain networks at baseline may serve as an individualized predictor of rTMS treatment response.

**Relevance statement:**

Dynamic reorganization of brain networks could predict the efficacy of repetitive transcranial magnetic stimulation in the spectrum of Alzheimer’s disease.

**Key points:**

• rTMS at the left angular gyrus could induce cognitive improvement.

• rTMS could suppress primary network connections to compensate for higher-order networks.

• Dynamic reorganization of brain networks could predict individual treatment response to rTMS.

**Graphical Abstract:**

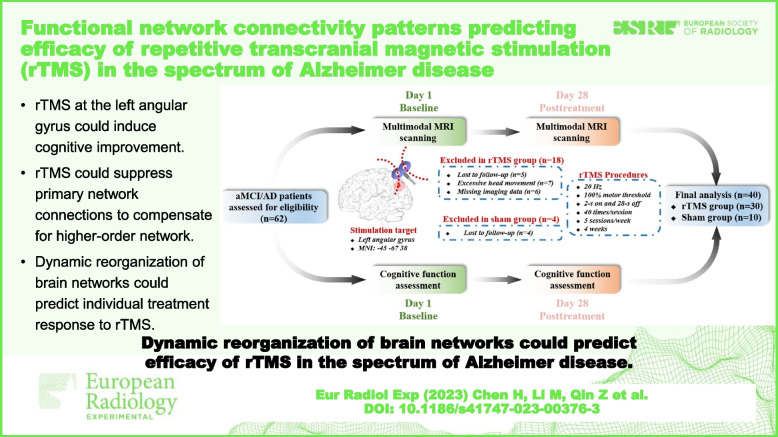

**Supplementary Information:**

The online version contains supplementary material available at 10.1186/s41747-023-00376-3.

## Background

Alzheimer’s disease (AD) is a globally prevalent debilitating degenerative disease marked by impaired memory function [1]. Repetitive transcranial magnetic stimulation (rTMS), as a new neuromodulation approach, could improve cognition in AD [2, 3]. However, the underlying mechanisms of rTMS interventions have not been well known.

Resting-state functional magnetic resonance imaging (rs-fMRI) is a method of studying the regional spontaneous neural activity and inter-regional functional connectivity to understand the underlying brain mechanisms [4]. AD showed altered functional connectivity mainly involving the default mode network (DMN) [5, 6]. However, these findings focused on the functional connectivity computed using rs-fMRI data of the entire scan, resulting in static brain connectivity that probably muddled together dynamic patterns of brain activity [7, 8]. Because potential abnormalities in brain dynamics are not identified, functional brain changes that characterize cognitive dysfunction are obscured. Recently, several studies have concentrated on revealing dynamic functional network connectivity (dFNC) in AD. Córdova-Palomera et al. [9] found decreased global metastability between functional states in which oscillatory modes were continuously changed. Another study [10] demonstrated that AD showed decreased dFNC in the sensorimotor network (SMN), visual network (VN), cerebellar network, and subcortical network.

rTMS is an effective tool for the treatment of various neuropsychological diseases [11]. The magnetic field can induce weak electric currents in cerebral regions, which generate stimulated and interconnected cortex neuroplasticity to regulate brain functions [12, 13]. An electroencephalography-TMS study indicated that an rTMS intervention improved episodic memory function by changing the connection between the precuneus and frontal lobe but had no positive effect on other cognitive domains [14]. In addition, Rutherford et al. [15] found that stimulating the bilateral dorsolateral prefrontal cortex significantly improved cognitive evaluation scores in AD patients. Ahmed et al. [16] further proposed that high-frequency rTMS (20 Hz) stimulated at the bilateral dorsolateral prefrontal cortex relative to low-frequency rTMS (1 Hz) could improve cognitive function in patients with mild to moderate AD. This therapeutic effect could be maintained for three months. In our previous studies [17, 18], we used the left angular gyrus as a stimulation target, although the mechanism by which its static functional network connectivity (sFNC) and dFNC induce cognition improvement is still largely unknown.

In this study, functional network analyses and machine learning methods were combined to reveal the underlying mechanisms of rTMS-related cognitive improvement and explore the predictive value of rTMS-related clinical efficacy based on the baseline functional network. We used the left angular gyrus as the stimulation target to explore the neural substrates of cognitive improvement by combining sFNC and dFNC analysis frameworks. Moreover, we hypothesized that the specific connectivity pattern at baseline could serve as a biomarker to predict the clinical efficacy of rTMS treatment using a support vector regression (SVR) model.

## Methods

### Participants

**Fig. 1:**
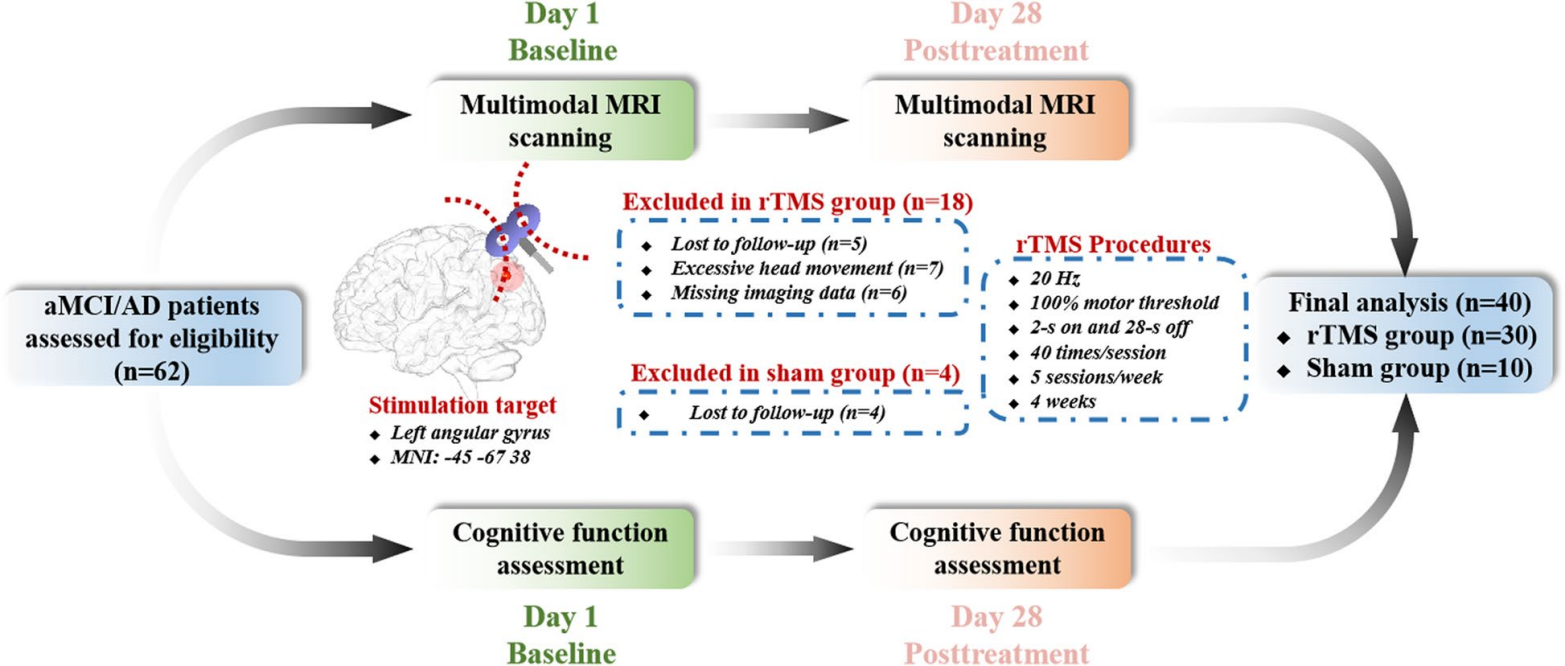
A summary of the study design and participant flow through the study

Sixty-two subjects with amnestic mild cognitive impairment (aMCI) and AD participated in this study. Due to aMCI and AD belonging to the AD spectrum disorder and the limited sample size in our study, we performed data analyses using combined data from AD and aMCI patients. The detailed inclusion and exclusion criteria were described in our previous studies and in the Supplementary Material [19–21]. All the participants were randomized to the rTMS or sham group (rTMS group: *n* = 48; sham group: *n* = 14) and fully blinded to allocation status (Fig. 1). In this study, aMCI and AD participants all took memantine or donepezil hydrochloride. We instructed participants to maintain their original medication regimen during the trial period. This study was approved by the ethics committee of our hospital (ChiCTR2100050496). All subjects gave written informed consent according to the Declaration of Helsinki.

### Neuropsychological measurement

The cognitive battery included general cognitive performance, memory function, executive function, language function, information processing speed, and visuospatial function, which are described in the Supplementary Material.

### MRI scanning

The multimodal neuroimaging data were acquired using a Philips Medical Systems 3.0 T machine. The detailed MRI protocol is described in the Supplementary Material.

### Neuronavigated rTMS procedure

rTMS was performed using the YIRUIDE CCY‐IV magnetic stimulator (Yiruide Co., Ltd., Wuhan, China) and Visor 2.0 neuronavigation system (Advanced Neuro Technologies, Enschede, the Netherlands). The left angular cortex (Montreal Neurological Institute coordinates: -45, -67, 38) was defined as the stimulation target based on the results of our previous cross-sectional experiment [17, 18]. The specific treatment parameters were consistent with our previous studies and described in the Supplementary Material [17, 18].

### Resting-state fMRI preprocessing

Rs-fMRI data were preprocessed by DPABI (http://www.rfmri.org/dpabi). The first 10 volumes were discarded to remove the equilibrium effect. The preprocessing included slice-timing correction, realignment, normalization, and spatially smoothing. Additionally, subjects with maximum head displacement higher than 2 mm, maximum rotation of more than 2°, or mean Jenkinson frame-wise displacement greater than 0.25 mm were excluded from this study.

### Group independent component analysis

**Fig. 2:**
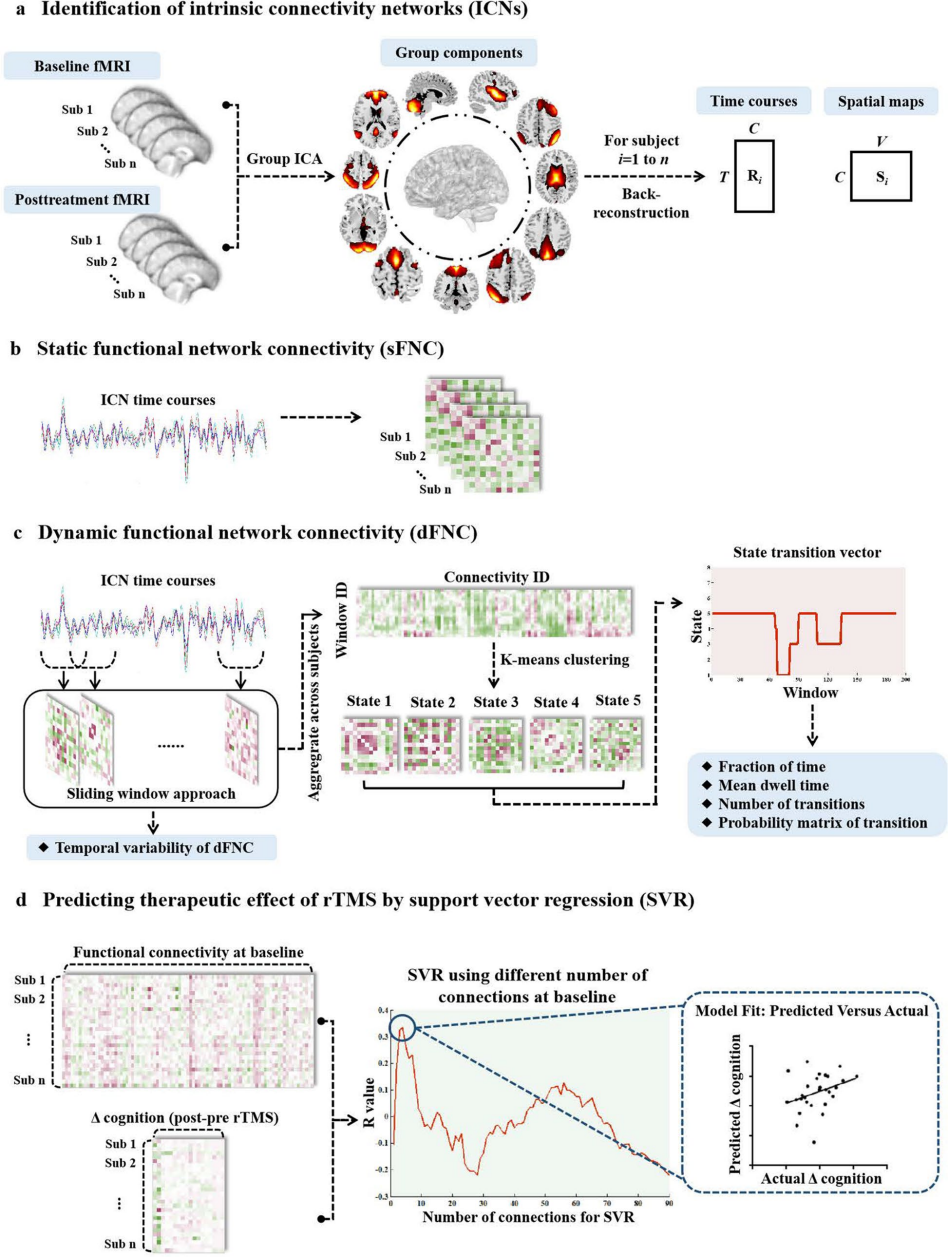
Flowchart of functional network connectivity analysis and predictive model construction. **a** Thirty-four ICs were obtained by GICA, then 14 ICs were assigned to fourteen brain networks. **b** sFNC is estimated as the pairwise correlation of the time courses. **c** dFNC is estimated by a sliding-window approach. K-means clustering is used to identify discrete dynamic connectivity states. We examined four different metrics based on the state transition vector, including fractional of time, mean dwell time, number of transitions, and probability matrix of transition. **d** Predicting the therapeutic effect of rTMS by SVR model. *ICs* Independent components, *GICA* Group independent component analysis, *sFNC* Static functional network connectivity, *dFNC* Dynamic functional network connectivity, *rTMS* Repetitive transcranial magnetic stimulation, *SVR* Support vector regression

**Fig. 3:**
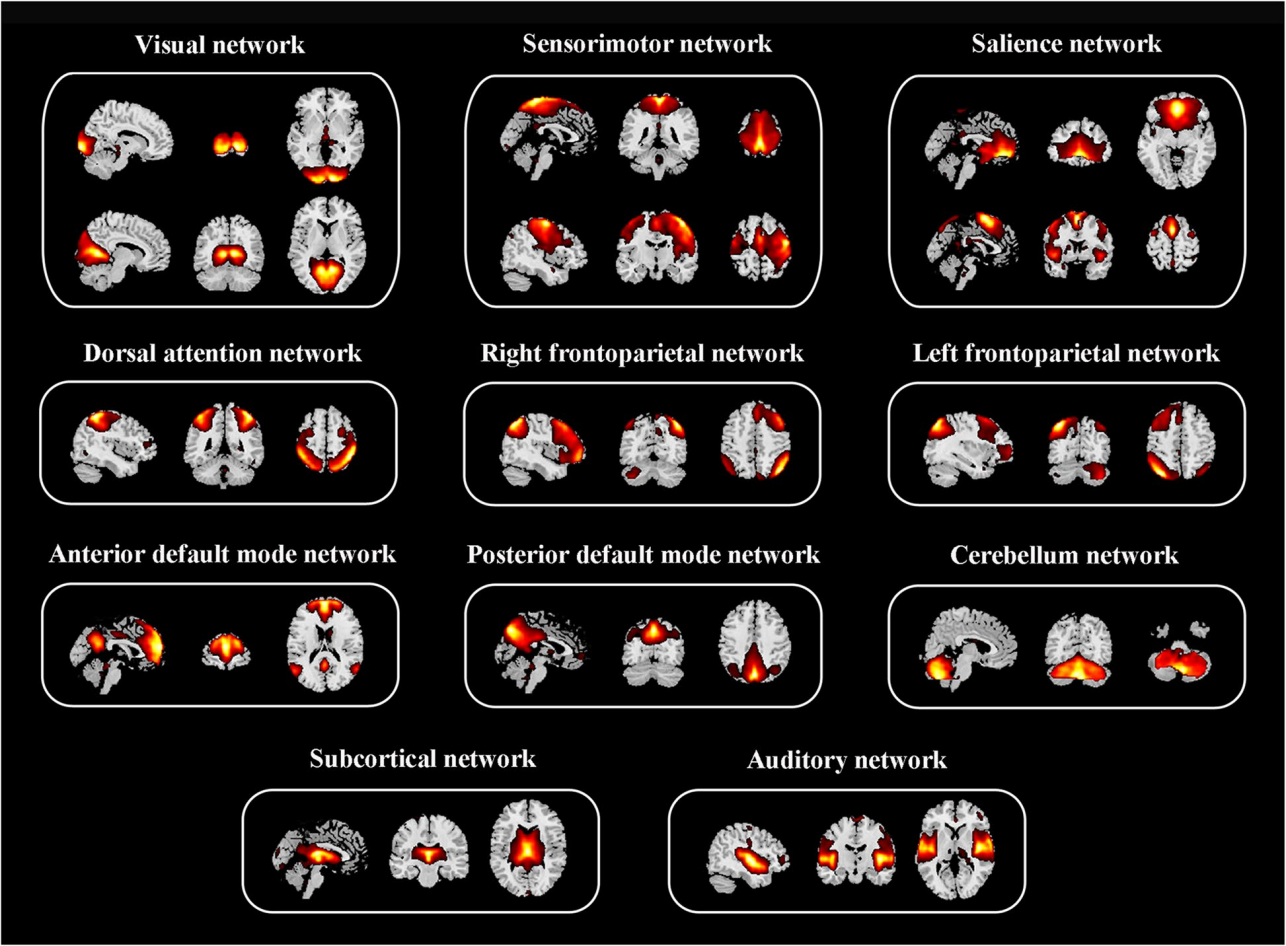
Spatial maps of 14 selected independent components

Group independent component analysis, conducted by GIFT v4.0b (http://mialab.mrn.org/software/gift/) (Fig. 2a), spatially decomposed all participants’ rs-fMRI data into a linear combination of independent components. Their number was estimated automatically based on the minimum description length method [22]. First, data dimension reduction was conducted to reduce the imaging data into 51 principal components analyzed by principal component analysis. Then, an infomax algorithm was applied to decompose the reduced data of all participants into an estimated 34 independent components [23]. To guarantee the repeatability of this estimation, this calculation process was repeated 20 times based on the ICASSO algorithm (https://research.ics.aalto.fi/ica/icasso/). Finally, time courses and spatial maps were obtained from the spatial–temporal back reconstruction approach [24]. The visual recognition and automatic identification method helped us select 14 functional networks: right and left frontoparietal networks (rFPN and lFPN), anterior and posterior DMN (aDMN and pDMN), medial and posterior VN (mVN and pVN), cerebellar network, dorsal attention networks, dorsal and ventral SMN (dSMN and vSMN), auditory network, salience network (SN1 and SN2), and subcortical network (Fig. 3).

### Static functional network connectivity (sFNC)

The sFNC analysis was conducted in GIFT software (Fig. 2b). sFNC was computed as the pairwise correlation between predefined 14 spatially independent components. Before FNC computation, the following processing procedures were conducted. Firstly, detrending, despiking detected outliers, and lowpass filtering were conducted. Then, the pairwise correlations between independent components were computed and Fisher’s *z*-transformation was used to improve the normality.

### Dynamic functional network connectivity (dFNC) and variability

The dFNC analysis was conducted as shown in Fig. 2c. A sliding window temporal approach was applied, in which the convolution of a window (window length = 30 TRs) with a Gaussian (*σ* = 3 TRs) was progressively refined, with sliding in steps of 1 TR. The correlation matrices of all windows converged to form a component × component × window matrix to represent temporal changes of FNC. The temporal variability of FNC was defined as the standard deviation of internetwork connectivity through all windows.

### State clustering analysis

A k-means algorithm was performed to cluster all windowed FNC matrices (Fig. 2c). First, we computed the FNC variability across all windows and selected the windows with local maxima in this connectivity variance as subject exemplars. Next, k-means clustering was conducted on these exemplars of all participants and repeated 150 times to obtain centroid states. The optimal number of clusters (*k* value) was determined according to the elbow criterion, which is computed as the ratio of within- to between-cluster distance. This number changed in the range of 2 to 10 to recognize the optimal value. Finally, the optimal *k* value in this study was estimated as *k* = 5 for further analysis, with each cluster representing a kind of functional connectivity state. Furthermore, we examined four state-related metrics: fractional of time, mean dwell time, number of transitions, and probability matrix of transition (detailed explanations were provided in the Supplementary Materials).

### Statistical analysis

Statistical analysis of demographic data and cognitive performance was performed by SPSS v22.0 software. The Shapiro–Wilk test was used to assess whether the data met the normal distribution and the Levene’s test was used to assess whether the data met homogeneity of variance. Differences in cognitive performance before and after stimulation were analyzed by paired *t* tests.

To investigate group differences of sFNC between pre- and post-rTMS treatment, paired *t* tests were performed (*p* < 0.05, uncorrected). Group differences in clustering-related metrics were also evaluated using paired *t* tests (*p* < 0.05, uncorrected). Pearson’s correlation analysis was performed to investigate the relationship between changes in FNC-related metrics and cognitive performance after rTMS treatment (*p* < 0.05, uncorrected).

### Individualized prediction of rTMS treatment effects

To explore whether the FNC at baseline could predict cognitive improvement after rTMS treatment, we performed SVR analysis implemented by the LIBSVM toolbox (http://www.csie.ntu.edu.tw/~cjlin/libsvm/index.html) Fig. 2d sFNC- and dFNC-related variables before rTMS treatment were respectively considered as features in the SVR model to predict the changed cognition (Δ cognition = cognition after rTMS minus cognition before rTMS). Δ cognition is defined as the the changed cognition performance after rTMS treatment. For example, Δ memory function is calculated by memory function after rTMS minus memory function before rTMS.

The leave-one-out cross-validation was applied to provide a conservative estimation of the prediction accuracy. Firstly, the sFNC array for each participant was converged to a feature vector. Secondly, the feature selection procedure was conducted by ranking features based on their correlation coefficient with Δ cognition (*p* < 0.05, uncorrected). Since the number of features affected the predictive accuracy, we increased one feature in each loop iteration based on the order of ranking. Thirdly, a predictive model was generated by SVR algorithm to explore the relation between the selected FNC features and Δ cognition in the training dataset. Fourthly, this model was applied to predict the Δ cognition of that test sample. In the case of leave-one-out cross-validation, a single subject’s predicted Δ cognition value is generated by taking the data from all other subjects as the training data set in an iterative manner until all subjects have a predicted Δ cognition value. The prediction performance was estimated by calculating Pearson’s correlations (*R* value) between observed and predicted Δ cognition scores. Due to a slightly different set of feature ranking in each leave-one-out cross-validation fold, consensus features were regarded as the common features always selected to form the final feature set. The same analysis steps as above were applied to elucidate the predictive value of the dynamic features for cognitive improvement after rTMS treatment.

## Results

### Demographic and clinical characteristics

In the rTMS group, eighteen subjects (6 aMCI and 12 AD patients) were excluded from analysis because of lost-to-follow-up (*n* = 5), excessive head movement (*n* = 7), and missing imaging data (*n* = 6). Additionally, two subjects (2 AD patients) were not included in the cognition analysis due to the incomplete cognition scale. In the sham group, four subjects (2 aMCI and 2 AD patients) were excluded from analysis due to lost-to-follow-up (*n* = 4) and ten subjects (10 aMCI) were included for imaging and cognition analyses. Finally, 30 subjects (21 aMCI and 9 AD patients) in the rTMS group were included for the imaging analyses and 28 subjects (21 aMCI and 7 AD patients) in the rTMS group were included for the cognition analyses (Fig. 1 and Table 1). Statistically significant improvements were seen after rTMS treatment in the MMSE (*t* = -2.48, *p* = 0.020), MoCA-BJ (*t* = -2.74, *p* = 0.011), memory function (*t* = -4.74, *p* < 0.001), and information processing speed (*t* = -2.17, *p* = 0.039) (Supplementary Fig. S1). In contrast, no significant difference in cognitive performance was found in the sham group (Supplementary Fig. S2).

**Table 1:** Demographic and neuropsychological data in the longitudinal experiment

Items	rTMS (*n* = 28)		*t/p*	Sham (*n* = 10)		*t/p*
	**Pre**	**Post**		**Pre**	**Post**	
**Demographics**						
Age (years)	66.00 ± 7.42		–	69.10 ± 6.71		–
Education (years)	11.71 ± 2.88		–	10.20 ± 1.55		–
Gender (male/female)	11/17		–	5/5		–
**General cognition**						
MMSE (raw score)	25.21 ± 5.13	26.32 ± 4.44	-2.48/0.02*	28.40 ± 1.27	28.50 ± 1.43	-0.26/0.80
MoCA-BJ (raw score)	20.86 ± 5.96	22.64 ± 5.38	-2.74/0.01*	23.50 ± 3.17	23.30 ± 3.37	0.21/0.84
**Multiple cognitive domain**						
**Memory function (*z* score)**	-0.27 ± 0.80	0.27 ± 0.98	-4.74/ < 0.001*	-0.06 ± 0.51	0.06 ± 0.87	-0.36/0.73
AVLT-DR (raw score)	3.32 ± 2.98	5.25 ± 3.47	-4.48/ < 0.001*	4.90 ± 1.45	5.20 ± 2.25	-0.46/0.66
VR-DR (raw score)	4.43 ± 3.98	6.68 ± 4.58	-3.76/0.001*	8.90 ± 2.28	9.10 ± 3.84	-0.17/0.87
**Visuospatial function (*z* score)**	-0.06 ± 1.05	0.06 ± 0.81	-1.14/0.23	-0.13 ± 1.04	0.13 ± 0.57	-0.89/0.40
CDT (raw score)	3.36 ± 1.16	3.43 ± 0.92	-0.40/0.69	3.60 ± 0.70	3.80 ± 0.42	-0.80/0.44
VR-C (raw score)	12.89 ± 2.42	13.32 ± 2.20	-2.00/0.06	13.40 ± 1.35	13.60 ± 1.27	-1.00/0.34
**Information processing speed (*z* score)**	-0.12 ± 0.74	0.12 ± 0.98	-2.17/0.04*	-0.08 ± 0.99	0.08 ± 0.22	-0.90/0.39
TMT-A (raw score)	78.29 ± 32.42	83.43 ± 50.01	-0.51/0.57	92.20 ± 59.28	73.30 ± 39.56	1.39/0.20
Stroop A (raw score)	25.36 ± 13.30	22.07 ± 10.43	1.90/0.07	26.80 ± 10.02	24.80 ± 7.29	0.94/0.37
Stroop B (raw score)	27.75 ± 11.47	26.32 ± 11.72	0.97/0.34	28.20 ± 10.43	24.60 ± 5.97	1.69/0.13
**Language function (*z* score)**	-0.15 ± 0.96	0.15 ± 0.77	-1.69/0.10	-0.14 ± 0.82	0.14 ± 1.02	-2.32/0.05
CVF (raw score)	15.07 ± 5.52	16.57 ± 4.38	-1.50/0.14	15.30 ± 3.40	18.00 ± 5.75	-2.32/0.05
BNT (raw score)	46.89 ± 11.92	50.21 ± 11.51	-1.40/0.17	47.10 ± 9.70	47.10 ± 9.70	–/–
**Executive function (*z* score)**	-0.10 ± 0.56	0.10 ± 0.97	-1.14/0.26	-0.06 ± 0.95	0.06 ± 0.61	-0.61/0.56
TMT-B (raw score)	175.46 ± 106.00	151.96 ± 131.34	1.15/0.26	206.60 ± 159.37	130.30 ± 54.04	1.75/0.11
Stroop C (raw score)	39.54 ± 14.24	47.46 ± 36.87	-1.31/0.20	37.70 ± 13.46	36.80 ± 14.35	0.21/0.84

Values are presented as the mean ± standard deviation (SD). The *p* value was obtained by paired *t* test. *Indicates a statistical difference between baseline and post-treatment, *p* < 0.05. *MMSE* Mini-mental state examination, *MoCA-BJ* Beijing version of the Montreal Cognitive Assessment, *AVLT-DR* Auditory verbal learning test-delayed recall, *VR-DR* Visual reproduction-delay recall, *CDT* Clock drawing test, *VR-C* Visual reproduction-copy, *CVF* Category verbal fluency, *BNT* Boston Naming Test, *TMT-A* and *TMT-B* Trail making test-A and B, *Stroop A, B and C* Stroop color and word tests A, B, and C

Additionally, we re-analyzed aMCI and AD data separately in the rTMS treatment group. In detail, we found that the aMCI subgroup (*n* = 21) showed cognitive improvement in the memory function (*t* = -4.19, *p* < 0.001), information processing speed (*t* = -2.53, *p* = 0.020), and the language function (*t* = -2.28, *p* = 0.034) after rTMS treatment. However, the treatment effect in the AD subgroup (*n* = 7) did not seem to be as good as that in the aMCI subgroup after the rTMS treatment (Supplementary Table 1).

### sFNC analysis and individualized prediction

**Fig. 4:**
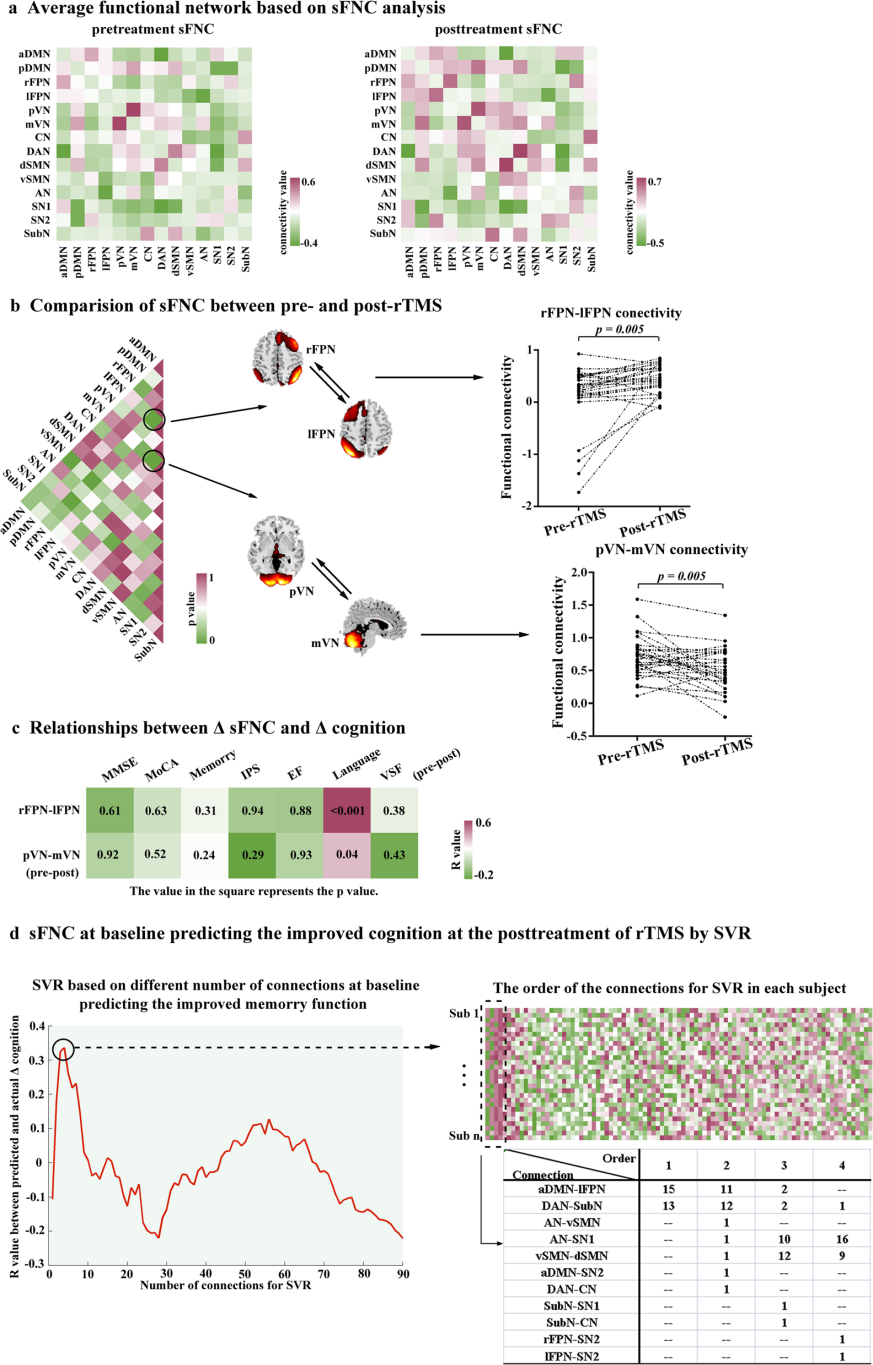
sFNC analysis and individualized prediction. **a** Average functional network based on sFNC analysis. **b** Comparison of sFNC between pre- and post-rTMS. Relative to pre-rTMS, participants after rTMS treatment exhibited significantly the increased connection between rFPN and lFPN (rFPN-lFPN connectivity, *p* = 0.005) and decreased connection between pVN and mVN (pVN-mVN connectivity, *p* = 0.005). **c** Relationship between Δ sFNC and Δ cognition. We observed a significantly negative correlation between Δ language function and Δ rFPN-lFPN connectivity (*r* = 0.64, *p* < 0.001), Δ pVN-mVN (*r* = 0.39, *p* = 0.042). **d** sFNC at baseline predicting the improved cognition at the posttreatment of rTMS by SVR. The linear SVR analysis achieved the best prediction ability (*R* = 0.37) when the 4 highest-ranked connections are applied. *sFNC* Static functional network connectivity, *dFNC* Dynamic functional network connectivity, *rTMS* Repetitive transcranial magnetic stimulation, *SVR* Support vector regression, *rFPN* Right frontoparietal networks, *lFPN* Left frontoparietal networks, *pVN* Posterior visual network, *mVN* Medial visual network

Figure 4a separately displays the group averaged sFNC matrices before and after rTMS treatment. Relative to pre-rTMS, participants after rTMS treatment exhibited significantly the increased connection between rFPN and lFPN (rFPN-lFPN connectivity, *p* = 0.005, uncorrected) and decreased connection between pVN and mVN (pVN-mVN connectivity, *p* = 0.005, uncorrected), as shown in Fig. 4b. Δ connectivity is defined as the changed connectivity after rTMS treatment (Δ connectivity = connectivity after rTMS minus connectivity before rTMS). We observed a significantly negative correlation between Δ language function and Δ rFPN-lFPN connectivity (*r* = 0.64, *p* < 0.001), Δ pVN-mVN (*r* = 0.39, *p* = 0.042) (Fig. 4c). While predicting the memory function after rTMS treatment, we found a significant predicted-observed correlation. As shown in Fig. 4d, the linear SVR analysis achieved the best prediction ability (*R* = 0.37) when the 4 highest-ranked connections are applied. The consensus functional connections identified in the cross-validation were mainly located across the aDMN and lFPN (Fig. 4d). In contrast, the sFNC patterns could not significantly predict other cognitive domains.

### dFNC analysis and individualized prediction

**Fig. 5:**
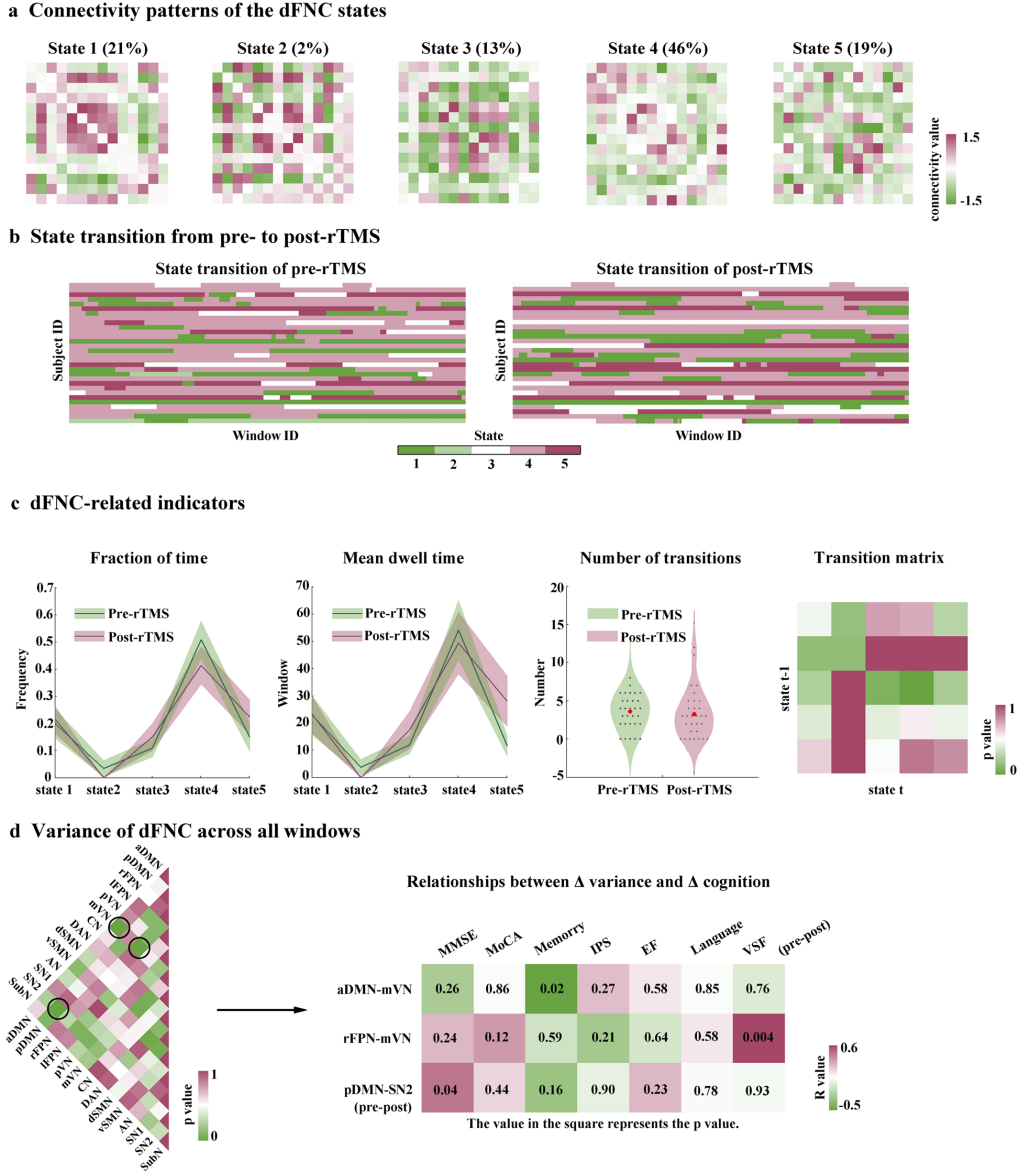
dFNC and state transition vector analysis. **a** Connectivity patterns of the dFNC states. **b** State transition from pre- to post-rTMS. The horizontal axis represents the window of the functional connectivity network and the vertical axis represents the included subjects. Five colors represent five different states (from state 1 to state 5). **c** Comparisons of state-related metrics. No significant differences were found in these state-related indicators. **d** Variance of dFNC across windows. The participants had increased dFNC variability between mVN and aDMN (*p* = 0.044, uncorrected), mVN and rFPN (*p* = 0.036, uncorrected), and decreased dFNC variability between pDMN and SN (*p* = 0.030, uncorrected) after rTMS treatment. The increased dFNC variability between mVN and rFPN was correlated with a changed VSF score (*r* = 0.52, *p* = 0.004). The Δ dFNC variability between mVN and aDMN was associated with Δ memory function (*r* = -0.45, *p* = 0.017), and the Δ dFNC variability between pDMN and SN was related to Δ MMSE (*r* = 0.40, *p* = 0.036). *dFNC* Dynamic functional network connectivity, *rTMS* Repetitive transcranial magnetic stimulation, *mVN* Medial visual network, *aDMN* Anterior default mode networks, *pDMN* Posterior default mode networks, *rFPN* Right frontoparietal networks, *SN* Salience network

The centroids and proportion of dFNC states are shown in Fig. 5a. The FNC patterns of State 4 resembled the posttreatment sFNC accounting for 46% of all windows, while state 5 showed similar patterns as the pretreatment sFNC accounting for 19%. By comparison, the FNC patterns of other states displayed connectivity diverging substantially from the sFNC patterns. Additionally, state 3 accounting for 13% showed densely connected intranetwork but loosely connected internetwork connectivity. A widely hyperconnected pattern was recognized in state 1 (21%), which is similar to state 2 (2%) except that state 2 displayed weak connectivity between FPN and other subnetworks.

The state transition vector of each participant from pre- to post-rTMS treatment is presented in Fig. 5b. The temporal properties of dFNC were computed in Fig. 5c. However, we did not observe any significant group differences in these state-related indicators. Figure 5d (left) showed the group difference of dFNC variability across windows between pre- to post-rTMS treatment. Briefly, both the participants had increased dFNC variability between mVN and aDMN (*p* = 0.044, uncorrected), mVN and rFPN (*p* = 0.036, uncorrected), and decreased dFNC variability between pDMN and SN (*p* = 0.030, uncorrected) after rTMS treatment. We found that the increased dFNC variability between mVN and rFPN was correlated with a changed VSF score (*r* = 0.52, *p* = 0.004). In addition, we also found the Δ dFNC variability between mVN and aDMN was associated with Δ memory function (*r* = -0.45, *p* = 0.017) and the Δ dFNC variability between pDMN and SN was related to Δ MMSE (*r* = 0.40, *p* = 0.036) (Fig. 5d). The SVR analysis could not significantly predict the rTMS effects based on these dFNC variabilities.

**Fig. 6:**
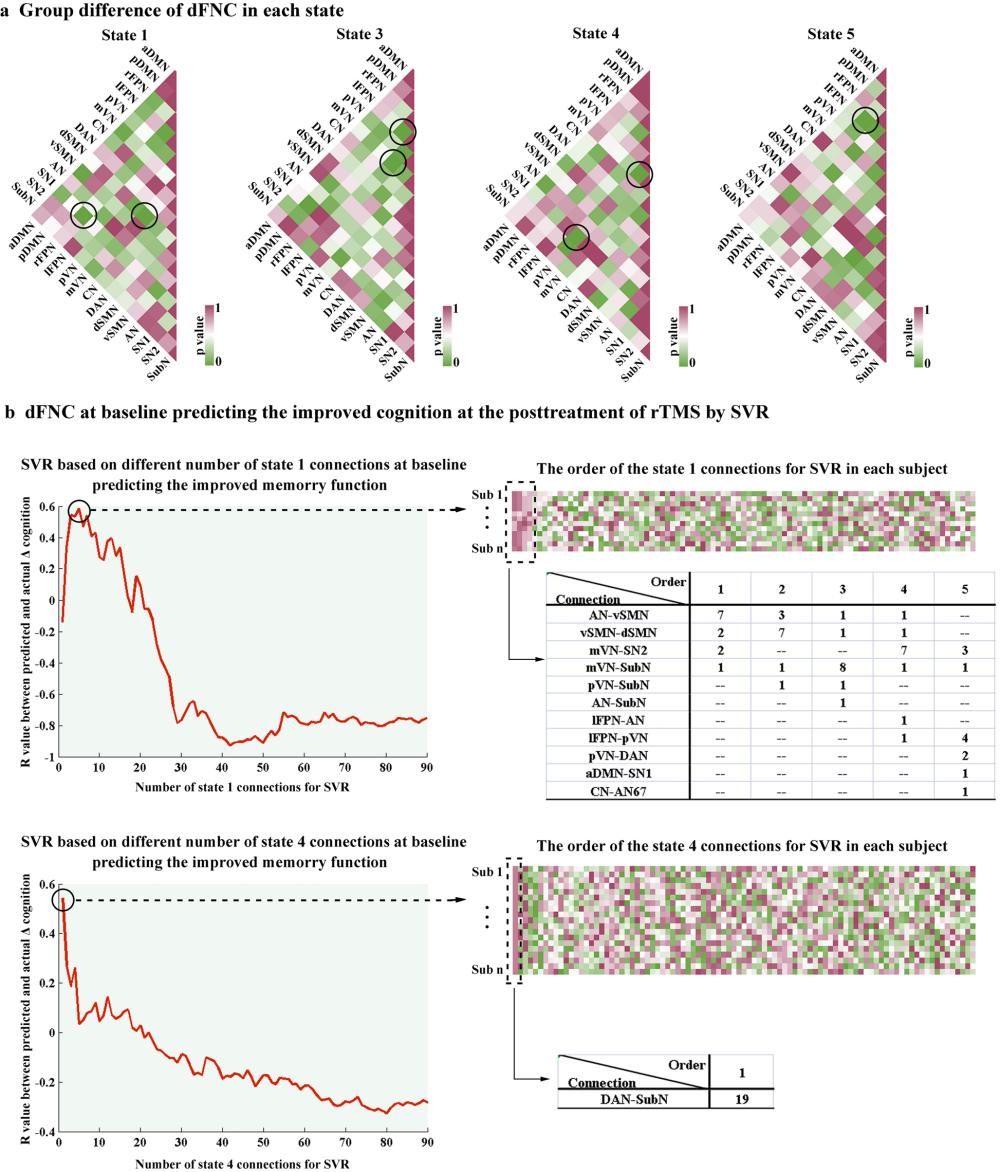
dFNC analysis in each state and individualized prediction. **a** Group differences of dFNC in each state. **b** dFNC at baseline predicting the improved cognition at the posttreatment of rTMS by SVR. The linear SVR analysis achieved the best prediction ability (*R* = 0.58) when the 5 highest-ranked functional connections of state 1 are applied. In addition, when the 1 highest-ranked connection of state 4 is applied, the linear SVR analysis achieved the best prediction ability of improved memory function (*R* = 0.54). *dFNC* Dynamic functional network connectivity, *rTMS* Repetitive transcranial magnetic stimulation, *SVR* Support vector regression

Within each state (except for state 2), participants exhibited abnormal transient dFNC patterns compared to the dFNC at baseline (Fig. 6a). In state 1, subjects after rTMS treatment had lower connectivity between rFPN and SN (*p* = 0.009, uncorrected), but higher connectivity between mVN and dSMN (*p* = 0.007, uncorrected). In state 3, we observed weaker connectivity between mVN and lFPN (*p* = 0.01, uncorrected) and stronger connectivity between rFPN and lFPN (*p* = 0.007, uncorrected) after rTMS treatment. In state 4, decreased connectivity within VN (*p* = 0.005, uncorrected) and between pVN and SN (*p* = 0.004, uncorrected) were found after posttreatment. In state 5, participants after rTMS treatment showed higher connectivity between rFPN and pDMN (*p* = 0.006, uncorrected).

When predicting the improved memory function based on dFNC after rTMS treatment, we found a significant predicted-observed correlation. As shown in Fig. 6b, the linear SVR analysis achieved the best prediction ability (*R* = 0.58) when the 5 highest-ranked functional connections of state 1 are applied. The consensus functional connections identified in the cross-validation were mainly located across the primary sensory network. Additionally, when the 1 highest-ranked connection of state 4 is applied, the linear SVR analysis achieved the best prediction ability of improved memory function (*R* = 0.54). The consensus functional connections identified in the cross-validation were mainly located between the dorsal attention network and the subcortical network. In contrast, the other dFNC state could not significantly predict rTMS efficacy.

## Discussion

In this study, the sFNC and dFNC were used to explore the underlying mechanism of network remodeling after rTMS intervention in AD spectrum disorder. Furthermore, machine learning methods were applied to predict the therapeutic efficacy of rTMS interventions through baseline connectivity. Our results showed that dynamic reorganization of the brain network (*e.g.*, suppressing primary network connections to compensate for higher-order cognitive network dysfunction) might be the mechanism of rTMS treatment shown by neuroimaging, and this regulation pattern could serve as a potential predictor of a favorable rTMS treatment response in AD.

As a complex system, the human brain is intrinsically organized into networks, producing a high degree of flexibility in the system [25, 26]. Our static analysis results showed that sFNC between the lFPN and rFPN was significantly enhanced, while sFNC within the VN was significantly weakened after the rTMS intervention. We found that higher sFNC between the frontoparietal networks was positively related to better language abilities. Previous research showed that functional connectivity between the frontal and parietal regions referring to the language network was enhanced when contextual cues were available to support language comprehension [27]. Accumulating evidence indicates that the FPN supports the comprehension of quantifiers and the relative contribution of parietal and prefrontal cortex activation is modulated by the quantifier class [28]. These results suggest that the FPN may be involved in linguistic processing, which is consistent with our findings.

We attempted to use baseline functional connectivity to predict rTMS treatment outcomes. The connectivity between the aDMN and lFPN could successfully predict memory function performance after rTMS treatment. Previous studies have found that the more participants can adapt flexibly to trial‐and‐error learning by enhancing the functional connectivity between the DMN and FPN, the greater they can learn via trial‐and‐error learning compared to error-less learning. Dynamic interactions between the DMN and FPN could enhance the effect of trial‐and‐error learning [29]. In addition, strengthened integration between networks is necessary for greater working memory performance [30, 31]. Notably, co‐activation between the DMN and FPN plays a significant role in the goal‐directed and introspective cognitive control associated with episodic memory [29, 32].

The FNC pattern during the entire rs-fMRI scan period is not static but dynamic. For all the windowed FNC matrices, we applied the k-means clustering methods to estimate reoccurring functional connectivity patterns (*i.e.*, connectivity state). The five functional states detected in this study could provide more information about the dynamic interactive relationships between functional networks than only focusing on the static pattern. Furthermore, individuals included in the current study spent more time in state 4 and state 1. The FNC in state 4 was characterized by intranetwork connections within the default mode network, visual network, and sensorimotor network. In contrast, state 1 was characterized by internetwork connections between functional connectivity networks.

The findings from our dFNC analyses did not simply mirror the sFNC results. After rTMS treatment, participants showed higher connectivity between the rFPN and pDMN in state 5. Previous studies have found that the connectivity strength between the DMN and FPN was positively associated with both visual and verbal creativity [33]. Additionally, we observed that after the rTMS treatment, participants exhibited weaker connectivity between the mVN and lFPN in state 3. In state 4, decreased connectivity within the VN and between the pVN and SN were found. Overall, these results revealed that an adaptive reconfiguration of large-scale brain networks might support cognitive function, which is in line with previous studies. It has been reported that higher-order cognitive networks, including the DMN, dorsal attention network and FPN, are mainly distributed in the association cortex area. The complicated interactions among higher-order functional networks support complex cognitive processes and behaviors. In addition, primary networks, including the SMN and VN, are mainly located in the unimodal cortex [34]. Our findings indicated that rTMS could generate adaptive, compensatory functional reorganization by suppressing primary network connections to compensate for higher-order cognitive network dysfunction in AD patients, which provided a novel perspective to reveal the neural mechanism of the rTMS treatment.

In addition, we assessed the potential utility of dFNC in the prediction of rTMS treatment outcomes. We found that the linear SVR analysis achieved the best prediction ability when the 5 highest-ranked functional connections of state 1 were applied. Moreover, when the highest-ranked functional connection of state 4 was used, the linear SVR analysis achieved the best prediction ability of improved memory function. Notably, recent studies have revealed that different dynamic metrics have differing abilities to predict cognitive function and individual reorganization and might reflect intrinsic properties of the brain and behavior [34]. This research further demonstrated that the use of rs-fMRI in combination with machine learning methods may be an effective approach to assess rTMS treatment outcomes and revealed the importance of dFNC.

We also found an interesting result that rFPN-mVN variation increased after the TMS treatment, which was positively correlated with visuospatial function. Currently, investigating the temporal variability of dFNC is believed to be an effective method to characterize intrinsic temporal fluctuations in functional connectivity and to associate network activities and behaviors [35, 36]. The fluctuation in connectivity over time could reflect brain network flexibility, which could be essential for cognitive abilities [37–39]. A recent study investigating the developmental changes in dFNC from childhood into adolescence found that higher functional variability with age may provide greater cognitive and behavioral flexibility [40, 41]. Together, these results may be explained by the prevailing theory: (1) higher brain variability reveals greater efficiency of information processing and (2) developmental improvement in cognitive function is undergirded by enhanced neural temporal dynamics [41–43]. This is consistent with our findings that higher connectivity variability between the rFPN and mVN is more conducive to cognitive function improvement.

The limitations of this study are as follows. First, patients with cognitive dysfunction often have different degrees of cortical brain atrophy, and the distance between the cortex and the scalp can significantly affect the TMS treatment effect. The potential effects of differences in local cortical thickness on TMS utility due to local brain atrophy were not fully considered in the study [44]. Second, this study was an exploratory study of participants from a single center. In the future, a multicenter clinical trial is needed. Third, the influence of noise such as the length of the sliding window and the number of states analyzed by k-means clustering on the dFNC analysis was not excluded. Fourth, due to aMCI and AD belonging to the AD spectrum disorder and the limited and unbalanced sample size in our study, we performed data analyses using combined data from AD and aMCI patients. We also try to reanalyze aMCI and AD data separately in the rTMS treatment group. The treatment effect in the AD subgroup did not seem to be as good as that in the aMCI subgroup after the rTMS treatment, which suggested the rTMS treatment as soon as possible in the early stage of AD. We look forward to expanding the sample size divided into subgroups to validate our results in future studies. Fifth, we did not perform multiple comparison corrections in this study. These findings should be considered exploratory and preliminary. Further studies in this area are needed to validate these findings.

In conclusion, our findings shed light on the dynamic regulation of brain network resources by demonstrating that rTMS could suppress primary network connections to compensate for higher-order cognitive network dysfunction in the spectrum of AD. Crucially, dynamic reorganization of brain networks at baseline may serve as a predictor of individual treatment response to rTMS.

### Supplementary Information

**Additional file 1:  Table S1.** Demographic and neuropsychological data in the rTMS treatment group. **Fig. S1.** Cognitive performance in the rTMS group before and after rTMS treatment. The rTMS group (*n* = 28) showed cognitive improvement in the memory function (t = -4.74, *p* < 0.001) and information processing speed (t = -2.17, *p* = 0.039) after rTMS treatment. **Fig. S2.** Cognitive performance in the sham rTMS group before and after rTMS treatment. No significant difference in cognitive performance before and after rTMS treatment was found in the sham group.

## Data Availability

The datasets used and/or analyzed during the current study are available from the corresponding author on reasonable request.

## Abbreviations

AD: Alzheimer’s disease
aMCI: Amnestic mild cognitive impairment
dFNC: Dynamic functional network connectivity
DMN: Default mode network
FPN: Frontoparietal network
rs-fMRI: Resting-state functional magnetic resonance imaging
rTMS: Repetitive transcranial magnetic stimulation
sFNC: Static functional network connectivity
SMN: Sensorimotor network
SN: Salience network
SVR: Support vector regression
VN: Visual network
