# Supplementary material for: Functional network connectivity patterns predicting the efficacy of repetitive transcranial magnetic stimulation in the spectrum of Alzheimer’s disease
Source: Eur Radiol Exp. 2023 Oct 24;7:63. doi: 10.1186/s41747-023-00376-3 (PMC10593644; doi:10.1186/s41747-023-00376-3)

**Functional network connectivity patterns predicting efficacy of repetitive transcranial magnetic stimulation in the spectrum of Alzheimer disease**

**ELECTRONIC SUPPLEMENTARY MATERIAL**

***Participants***

The possible or probable AD was diagnosed based on the National Institute of Neurological and Communicative Disorders and Stroke and the AD and Related Disorders Association (NINCDS-ADRDA) and the Diagnostic and Statistical Manual of Mental Disorders IV criteria (DSM-IV) guidelines. Other key inclusion criteria included the scores for the Mini-Mental State Examination (MMSE) < 24 and clinical dementia rating (CDR) score 1 or 2. The MCI patients included in this study were diagnosed according to the recommendations of Petersen and described as follows: (1) memory complaint confirmed by the subject and/or an informant; (2) objective cognitive performance documented by an auditory verbal learning test-delayed recall (AVLT-DR) scores below or equal to 1.5 SD of education- and age-adjusted norms; (3) CDR score = 0.5; (4) MMSE scores ≥ 24; and (5) not sufficient to dementia according to NINCDS-ADRDA and DSM-IV. More detailed information about the criteria of aMCI has been described in our previous study. The HC subjects were required to have MMSE scores ≥ 26 and CDR score of 0. In addition, subjects with a history of other psychiatric or neurological disease (e.g., stroke, Parkinson’s disease, depression, traumatic brain injury, and others) were excluded in this study.

***Neuropsychological measurement***

In the current study, the neurocognitive assessment was administered before treatment and at the endpoint (pre- and post-rTMS). The cognitive battery comprised the following tests: *general cognitive performance* - Mini-Mental State Examination (MMSE) and the Beijing version of the Montreal Cognitive Assessment (MoCA-BJ); *memory function*: the auditory verbal learning test-delayed recall (AVLT-DR) and visual reproduction-delayed recall (VR-DR); *executive function*: Trail Making Test-B (TMT-B) and Stroop Color and Word Tests C (Stroop C); *language function*: Category Verbal Fluency (CVF) and Boston Naming Test (BNT); *information processing speed*: TMT-A, Stroop A and Stroop B; *visuospatial function*: visual reproduction-copy (VR-C) and Clock Drawing Test (CDT). To construct the composite z score, variables with smaller values representing better performance (e.g., TMT-A, TMT-B, Stroop A, Stroop B and Stroop C) were first divided by 1 so that for all variables, larger values implied better performance. Second, all measures were transformed to standardized z scores. Finally, the cognitive composite score of each domain, which served as the neuropsychological outcome variable, was respectively calculated as the mean of the corresponding sub-test scores.

***MRI scanning***

The multimodal neuroimaging data were acquired using a Philips Medical Systems 3.0T machine. Multimodal MRI scans were acquired before and after stimulation. The protocol included the high-resolution 3DT1-weighted imaging [echo time = 4.6 ms, repetition time = 9.8 ms, ﬂip angle = 8°, field of view = 250×250 mm^2^, acquisition matrix = 256×256, number of slices = 192, thickness = 1.0 mm] and a gradient-recalled echo planar imaging sequence (time of repetition [TR] = 2000 ms, ﬂip angle = 90°, echo time = 30 ms, number of slices = 35, acquisition matrix = 64 × 64, field of view = 240 × 240 mm^2^, thickness = 4 mm). During the MRI scanning, all subjects were asked to close their eyes and not fall asleep.

***Neuronavigated rTMS procedure***

rTMS was performed using the YIRUIDE CCY‐IV magnetic stimulator. The specific treatment parameters of this study were 20 Hz, 40 trains of 2 sec, 28-sec inter-interval, 1600 pulses/session and 100% resting motor threshold. This trial lasted 4 weeks including 20 daily rTMS sessions with 2 days off each weekend. The left angular cortex (MNI coordinates [-45, -67, 38]) is defined as the stimulation target. To accurately target the coil placement, a sphere of 6-mm radius based on this target site was converted to each participant’s 3DT_1_ by applying an inverse transformation produced during 3DT_1_ segmentation in SPM12 (http://www.fil.ion.ucl.ac.uk/spm/software/spm12/) and TMS target software (http://www.brainhealthy.net). Then, the sphere target of each participant in the individual space was entered into the Visor 2.0 neuronavigation system. In the sham group, the same stimulation parameter was used with the placebo coil positioned over the same target area but did not induce a treatment effect.

**State-related metrics**

We examined the temporal properties of dynamic functional connection states with four different variables. The fractional of time is the proportion of time spent in each state as measured by percentage. The mean dwell time represents how long the participant stayed in a certain state, which was calculated by averaging the number of consecutive windows belonging to one state before changing to other states. The number of transitions represents how many times either state changed from one to another, counting the number of times a switch occurred, with more transitions representing less stability over time. The probability matrix of transition is the probability of the switch from state i at time m to state j at time m + 1.

**Supplementary Table S1**

| **Demographic and neuropsychological data in the rTMS treatment group** | | | | | | |
| --- | --- | --- | --- | --- | --- | --- |
|  |  |  |  |  |  |  |
| **Items** | **aMCI (n = 21)** | | ***t/p*** | **AD (n = 7)** | | ***t/p*** |
|  | **Pre** | **Post** |  | **Pre** | **Post** |  |
| **Demographics** |  |  |  |  |  |  |
| Age (years) | 66.24±7.84 | | -- | 65.29±6.53 | | -- |
| Education (years) | 12.14±2.54 | | -- | 10.43±3.65 | | -- |
| Gender (male/female) | 9/12 | | -- | 2/5 | | -- |
| **General cognition** | | | | | | |
| MMSE (raw score) | 27.67±1.77 | 28.24±1.79 | -1.19/0.25 | 17.86±4.88 | 20.57±5.16 | -3.24/0.02* |
| MoCA-BJ (raw score) | 23.48±3.42 | 24.81±3.12 | -1.70/0.11 | 13.00±4.97 | 16.14±2.13 | -3.04/0.02* |
| **Multiple cognitive domain** | | | | | | |
| **Memory Function (z-score)** | 0.01±0.72 | 0.61±0.83 | -4.19/<0.001* | -1.11±0.27 | -0.73±0.67 | -2.27/0.06 |
| AVLT-DR (raw score) | 4.14±2.85 | 6.19±3.20 | -3.67/0.002* | 0.86±1.86 | 2.43±2.76 | -3.67/0.01* |
| VR-DR (raw score) | 5.81±3.63 | 8.38±3.76 | -3.61/0.002* | 0.29±0.76 | 1.57±2.57 | -1.19/0.28 |
| **Visuospatial Function (z-score)** | 0.31±0.26 | 0.34±0.35 | -0.60/0.56 | -1.18±1.66 | -0.77±1.21 | -1.09/0.32 |
| CDT (raw score) | 3.76±0.44 | 3.76±0.54 | -0.001/1.00 | 2.14±1.77 | 2.43±1.13 | -0.42/0.69 |
| VR-C (raw score) | 13.71±0.64 | 13.86±0.48 | -1.37/0.19 | 10.43±3.95 | 11.71±4.11 | -1.72/0.14 |
| **Information Processing Speed (z-score)** | 0.05±0.72 | 0.39±0.95 | -2.53/0.02* | -0.63±0.61 | -0.70±0.49 | 0.68/0.52 |
| TMT-A (raw score) | 72.48±27.27 | 66.29±29.96 | 1.29/0.21 | 95.71±42.19 | 134.86±64.40 | -1.26/0.26 |
| Stroop A (raw score) | 21.76±6.85 | 20.00±8.85 | 1.03/0.32 | 36.14±21.43 | 28.29±12.97 | 1.76/0.13 |
| Stroop B (raw score) | 26.57±11.69 | 23.38±10.22 | 2.13/0.05 | 31.29±10.83 | 35.14±12.20 | -1.22/0.27 |
| **Language Function (z-score)** | 0.13±0.64 | 0.39±0.53 | -2.28/0.03* | -0.97±1.30 | -0.59±0.94 | -0.60/0.57 |
| CVF (raw score) | 16.52±4.36 | 17.86±3.95 | -1.29/0.21 | 10.71±6.65 | 12.71±3.35 | -0.75/0.48 |
| BNT (raw score) | 49.90±7.22 | 52.95±1.19 | -3.14/0.005* | 37.86±10.41 | 42.00±19.81 | -0.43/0.68 |
| **Executive Function (z-score)** | -0.04±0.60 | 0.33±0.99 | -1.73/0.10 | -0.27±0.44 | -0.59±0.43 | 2.14/0.08 |
| TMT-B (raw score) | 179.14±118.94 | 143.43±142.73 | 1.68/0.11 | 164.43±56.74 | 177.57±93.24 | -0.25/0.81 |
| Stroop C (raw score) | 38.10±13.06 | 37.33±13.99 | 0.22/0.83 | 43.86±17.74 | 77.86±58.15 | -1.72/0.14 |
| Values are presented as the mean ± standard deviation (SD). | | | | | | |
| The *p* value was obtained by paired *t*-test. | | | | | | |
| * indicates a statistical difference between basline and posttreatment, *p* < 0.05. | | | | | | |
| *AD* Alzheimer disease, *aMCI* amnestic mild cognitive impairment, *rTMS* repetitive transcranial magnetic stimulation, *MMSE* mini mental state examination, *MoCA-BJ* Beijing version of the Montreal Cognitive Assessment, *AVLT-DR* Auditory Verbal Learning Test-delayed recall, *VR-DR* Visual reproduction-delay recall, *CDT* Clock Drawing Test, *VR-C* Visual reproduction-copy, *CVF* ategory verbal fluency, *BNT* Boston Naming Test, *TMT-A* and *TMT-B* Trail Making Test-A and B, Stroop A, B and C, Stroop Color and Word Tests A, B, and C. | | | | | | |

The aMCI subgroup (*n* = 21) showed cognitive improvement in the memory function, information processing speed and the language function after rTMS treatment. However, the treatment effect in the AD subgroup didn’t seem to be as good as that in the aMCI subgroup after the rTMS treatment. Abbreviations: AD, Alzheime r disease; aMCI, amnestic mild cognitive impairment; rTMS, repetitive transcranial magnetic stimulation; MMSE, mini mental state examination; MoCA-BJ, Beijing version of the Montreal Cognitive Assessment; AVLT-DR, Auditory Verbal Learning Test-delayed recall; VR-DR, visual reproduction-delay recall; CDT, Clock Drawing Test; VR-C, visual reproduction-copy; CVF, category verbal fluency; BNT, Boston Naming Test; TMT-A and TMT-B, Trail Making Test-A and B; Stroop A, B and C, Stroop Color and Word Tests A, B, and C.

**Supplementary Fig. S1**: Cognitive performance in the rTMS group before and after rTMS treatment. The rTMS group (n = 28) showed cognitive improvement in the memory function (*t* = -4.74, *p* < 0.001) and information processing speed (*t* = -2.17, *p* = 0.039) after rTMS treatment.

**
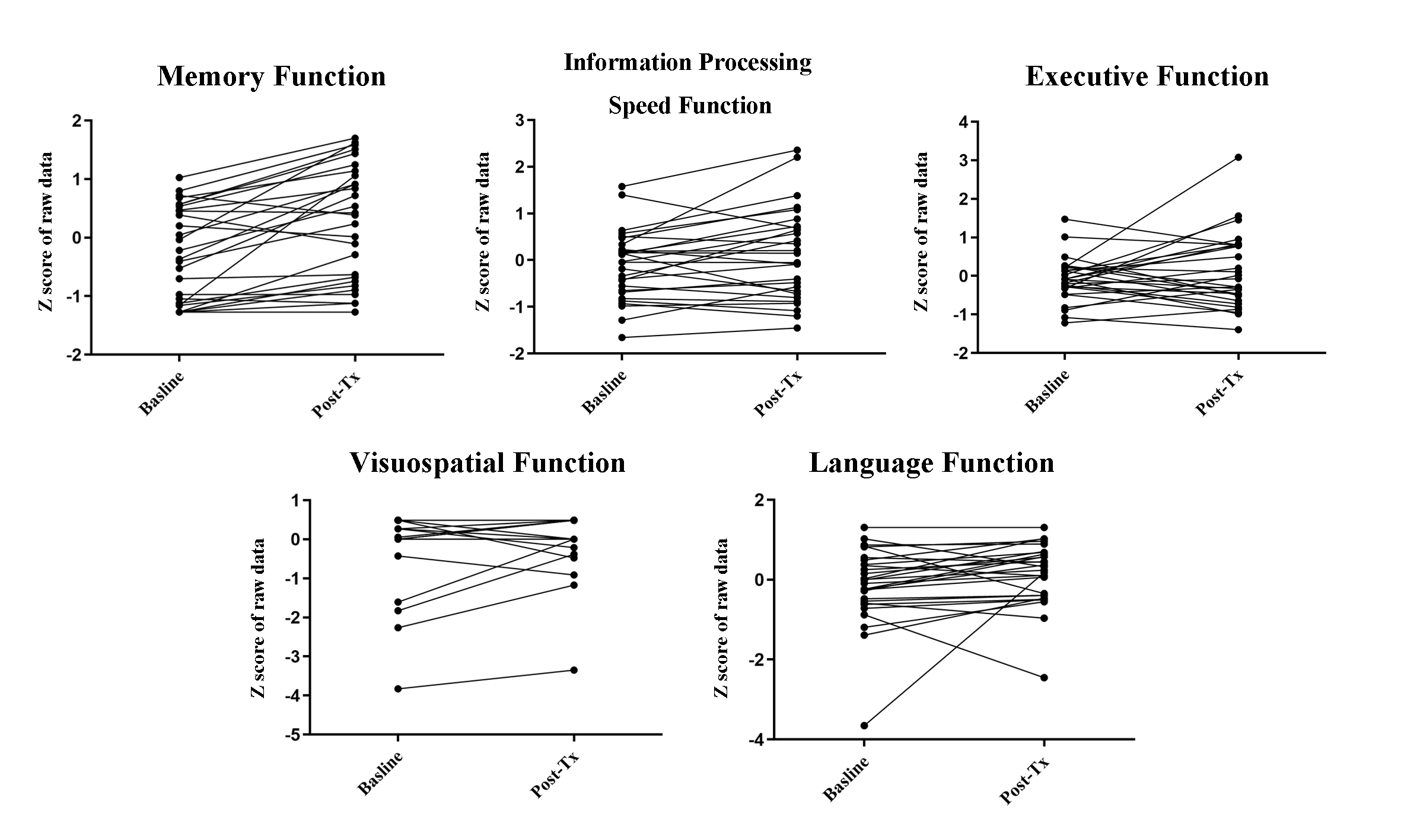
**

**Supplementary Fig. S2**: Cognitive performance in the sham rTMS group before and after rTMS treatment. No significant difference in cognitive performance before and after rTMS treatment was found in the sham group.


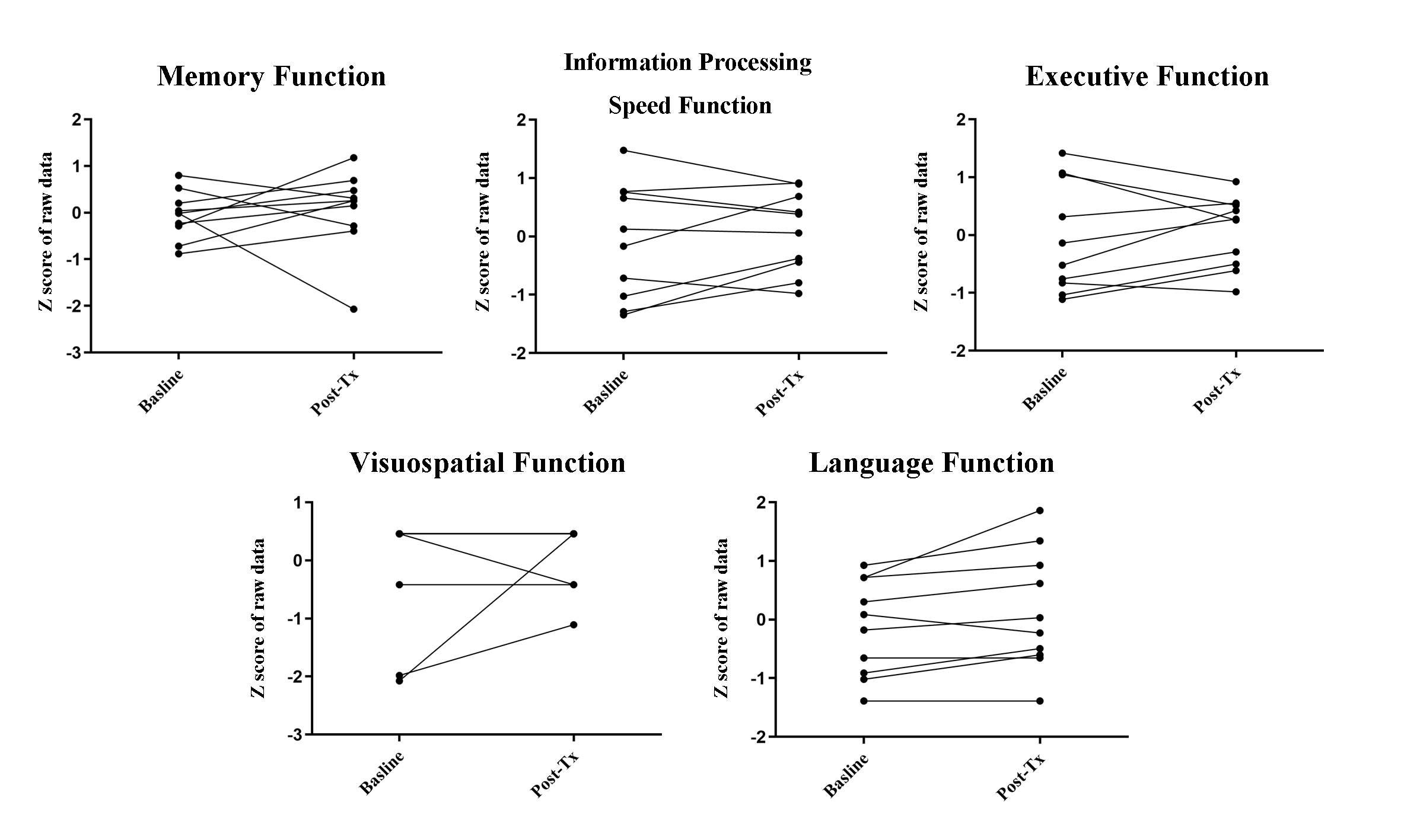

Supplement: Supplementary file 1 — Additional file 1: Table S1. Demographic and neuropsychological data in the rTMS treatment group. Fig. S1. Cognitive performance in the rTMS group before and after rTMS treatment. The rTMS group (n = 28) showed cognitive improvement in the memory function (t = -4.74, p < 0.001) and information processing speed (t = -2.17, p = 0.039) after rTMS treatment. Fig. S2. Cognitive performance in the sham rTMS group before and after rTMS treatment. No significant difference in cognitive performance before and after rTMS treatment was found in the sham group. [file 41747_2023_376_MOESM1_ESM.doc]
